# N-Terminal Segment of *Tv*CyP2 Cyclophilin from *Trichomonas vaginalis* Is Involved in Self-Association, Membrane Interaction, and Subcellular Localization

**DOI:** 10.3390/biom10091239

**Published:** 2020-08-26

**Authors:** Sarita Aryal, Hong-Ming Hsu, Yuan-Chao Lou, Chien-Hsin Chu, Jung-Hsiang Tai, Chun-Hua Hsu, Chinpan Chen

**Affiliations:** 1 Institute of Biomedical Sciences, Academia Sinica, Taipei 115, Taiwan; sarita2@ibms.sinica.edu.tw (S.A.); yclou@ibms.sinica.edu.tw (Y.-C.L.); therion@gate.sinica.edu.tw (C.-H.C.); taijh@gate.sinica.edu.tw (J.-H.T.); 2 Chemical Biology and Molecular Biophysics, Taiwan International Graduate Program, Academia Sinica, Taipei 115, Taiwan; 3 Department of Chemistry, National Tsinghua University, Hsinchu 300, Taiwan; 4 Department of Tropical Medicine and Parasitology, National Taiwan University, Taipei 106, Taiwan; hongming@ibms.sinica.edu.tw; 5 Department of Agricultural Chemistry, National Taiwan University, Taipei 106, Taiwan

**Keywords:** Cyclophilin, cytoadherence, peptidyl-prolyl isomerase, protein trafficking, *Trichomonas vaginalis*, trichomoniasis

## Abstract

In *Trichomonas vaginalis* (*T. vaginalis*), cyclophilins play a vital role in dislodging Myb proteins from the membrane compartment and leading them to nuclear translocation. We previously reported that *Tv*CyP1 cyclophilin from *T. vaginalis* forms a dimer and plays an essential role in moving the Myb1 transcription factor toward the nucleus. In comparison, *Tv*CyP2 containing an extended segment at the N-terminus (N-terminal segment) formed a monomer and showed a different role in regulating protein trafficking. Four X-ray structures of *Tv*CyP2 were determined under various conditions, all showing the N-terminal segment interacting with the active site of a neighboring *Tv*CyP2, an unusual interaction. NMR study revealed that this particular interaction exists in solution as well and also the N-terminal segment seems to interact with the membrane. In vivo study of *Tv*CyP2 and *Tv*CyP2-∆N (*Tv*CyP2 without the N-terminal segment) indicated that both proteins have different subcellular localization. Together, the structural and functional characteristics at the N-terminal segment offer valuable information for insights into the mechanism of how *Tv*CyP2 regulates protein trafficking, which may be applied in drug development to prevent pathogenesis and disease progression in *T. vaginalis* infection.

## 1. Introduction

Cyclophilins (CyPs) are a family of ubiquitous and versatile enzymes found in mammals, bacteria, plants, insects, and fungi [1]. Besides peptidyl-prolyl isomerase (PPIase) activity that catalyzes the interconversion of *cis/trans* isomerization of prolyl peptide bonds [2], CyPs possess multiple biological functions, including protein folding and trafficking, immune response, signal transduction, viral infection, and transcription regulation [1,3,4].

In humans, 24 unique CyPs have been identified [5,6]. Human cyclophilin A (*h*CyPA), a highly abundant protein that makes up 0.1–0.6% of the total cytosolic proteins [7,8], is the extensively studied cyclophilin. *h*CyPA initially is the primary cytosolic binding protein of cyclic undecapeptide cyclosporine A (CsA), an immunosuppressive drug [9,10]. The *h*CypA–CsA complex can inhibit the phosphatase activity of calcineurin [11,12], which prevents the transcription of genes encoding cytokines and subsequently suppresses interleukin 2 expression [13,14]. More importantly, *h*CyPA may be a potential target for antiviral therapy because it interacts with viral capsids, such as SARS-CoV [15,16,17] and HIV-1 [18,19], and plays a key factor in virus replication [20]. In recent months, the novel coronavirus COVID-19 outbreak has caused a severe pandemic, with more than 17,084,446 infected and 668,250 killed worldwide as of July 30, 2020 [21]. The outbreak further demonstrates the importance and urgent need for more research into *h*CyPA and perhaps other CyPs for developing antiviral drugs.

*Trichomonas vaginalis* (*T. vaginalis*) is an anaerobic and flagellated protozoan parasite that causes trichomoniasis, one of the common non-viral yet overlooked sexually transmitted infections [22,23,24]. This human pathogen also elevates the risk of cervical and prostate cancers and infertility and increases susceptibility to HIV and human papillomavirus transmission [25,26,27,28,29]. In *T. vaginalis* infection, parasitic growth and disease progression are related to a contact-dependent mechanism called cytoadherence [30]. A hydrogenosomal malic enzyme, encoded by the *ap65-1* gene primarily involved in carbohydrate metabolism, serves as a marker for hydrogenosome [31], and the transcription of this gene is mediated by the coordinated action of Myb1, Myb2, and Myb3 transcription factors [32,33,34].

**Figure 1:**
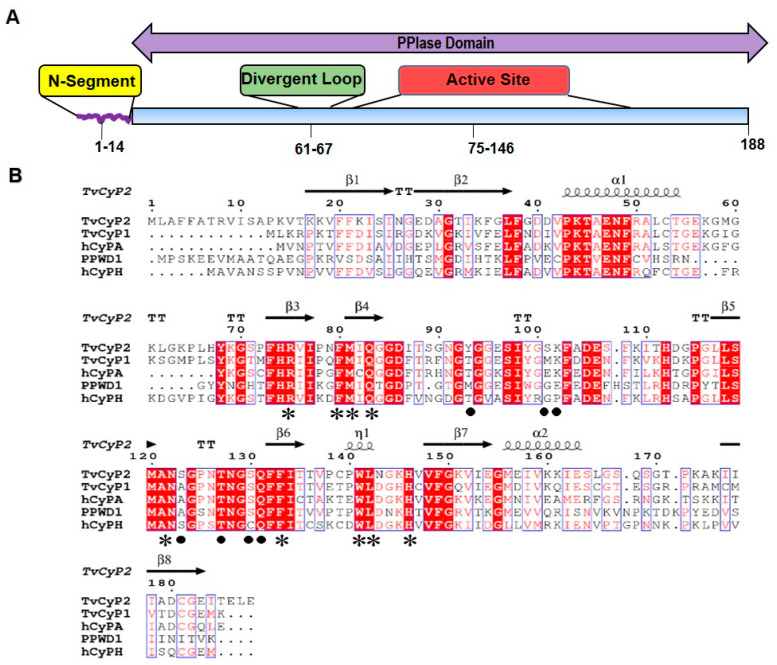
(**A**) A schematic representation showing the positions of N-terminal segment, divergent loop and the active site in *T. vaginalis* cyclophilin 2 (*Tv*CyP2). (**B**) Sequence alignments of *Tv*CyP2 with the cyclophilins mentioned in this article, including *Tv*CyP1, human cyclophilin A (*h*CyPA), *h*PPWD1, and *h*CyPH, which share 69%, 64%, 48%, and 56% sequence identity with *Tv*CyP2, respectively. The conserved residues are shown in white with a red background and similar residues in red with white background. The secondary structures of *Tv*CyP2 are on top of the sequence, and residues in S1 pocket (active site) and S2 pocket are indicated by an asterisk and circle, respectively, on the bottom of the sequence. The alignment was generated by using Clustal Omega and ESPRIPT.

Previously, we reported that *T. vaginalis* cyclophilin 1 (*Tv*Cyp1), a cyclophilin type peptidyl-prolyl isomerase and homolog of *h*CyPA, plays important roles in regulating the nuclear translocation of Myb1 and Myb3 proteins and forms a homodimer in both crystal and solution states with the active site residues highly exposed [35]. As compared with *Tv*Cyp1, *Tv*CyP2, containing an extra segment at the N-terminus (N-terminal segment), as shown in Figure 1A,B, is found primarily in the endoplasmic reticulum (ER) and regulates protein trafficking of *Tv*CyP1 and Myb3 toward hydrogenosomes and also *Tv*CyP1 toward the plasma membrane [36]. Apparently, both *Tv*Cyp1 and *Tv*CyP2 exhibit different mechanisms in regulating protein trafficking. Hence, we need information on the structural features of *Tv*CyP2 for further insights into why *Tv*Cyp1 and *Tv*CyP2 have different properties in regulating protein trafficking.

In this work, we initially performed an NMR experiment to investigate whether *Tv*CyP2 is catalytically active by using a Myb3_52–59_ peptide (ENGPQNWP) that contains a Gly-Pro bond as a substrate. X-ray crystal structure of *Tv*CyP2 showed that in addition to a typical cyclophilin tertiary fold, the N-terminal segment of *Tv*CyP2 interacted with the active site of an adjacent *Tv*CyP2. Further NMR studies indicated that this interaction also occurred in solution and the N-terminal segment likely interacts with the membrane. In vivo functional study of *Tv*CyP2 and *Tv*CyP2 without the N-terminal segment (*Tv*CyP2-∆N) revealed that both proteins have different subcellular localization. The structural and functional features of the N-terminal segment in *Tv*CyP2 provide important information for better understanding of how *Tv*CyP2 regulates protein trafficking, which may help in drug development for combating trichomoniasis, the disease caused by *T. vaginalis*.

## 2. Materials and Methods

### 2.1. Construction of Plasmids

Plasmids construction and transfectants establishment for the hemoaglutinin (HA)-tagged *Tv*CyP2 and *Tv*CyP2 (R75A) were performed as previously described [36]. To produce the N-terminal deletion of *Tv*CyP2, the DNA fragment amplified from pFLP-HA-*Tv*CyP2 by a primer pair, *Tv*CyP2(dN)-5′(5′GGATCCATGAAGGTCACAAAGAAAGTCTTC3′) and Sp6 primer, was digested by *BamH*I*/Xho*I, and then ligated into *BamH*I*/Xho*I–restricted pFLP-HA-*Tv*CyP2, to generate plasmid of pFLP-HA-*Tv*CyP2 (dN). The negative controls were the non-transfected cells.

### 2.2. Preparations of Recombinant Proteins and Peptides

*Tv*CyP2 and *Tv*CyP2-∆N were cloned into the pET-29b vector (Novagen, Darmstadt, Germany) by using NdeI and XhoI restriction sites and were expressed in *E. coli* strain BL21(DE3) with an additional His-tag at the C-terminus [37]. The cloned *Tv*CyP2-∆N consists of an extra Met residue at the N-terminus. For *Tv*CyP2 and *Tv*CyP2-∆N unlabeled samples, *E. coli* cells were cultured in lysogenic broth medium at 37 °C, and protein synthesis was induced after 3–4 h of growth by the addition of 1 mM isopropyl β-D-1-thiogalactopyranoside (IPTG) with the culture medium until reaching optical density (OD) 600 nm of 0.5–0.6. The cell pellets were then resuspended in 20 mM NaH_2_PO_4_, 500 mM NaCl at pH 7.0, and lysed by using an M-110S microfluidizer (Microfluidics, Newton, MA, USA). The lysed cells were centrifuged (Avanti J-26 XP, Ramsey, MN, USA) at 17418 rcf for 45 min to separate the cytosolic fraction from the insoluble fraction. To purify *Tv*CyP2 and *Tv*CyP2-∆N, supernatant from the lysate was passed through the anion exchange resin (Q-sepharose fast flow, GE healthcare, Uppsala, Sweden) and then flow-through was loaded to nickel-nitrilotriacetic acid (Ni-NTA) affinity resin (Qiagen, Hidden, Germany) equilibrated with 20 mM NaH_2_PO_4_, 500 mM NaCl, and 0.3 mM NaN_3_ at pH 7.0. *Tv*CyP2 and *Tv*CyP2-∆N were eluted at 200 mM imidazole, 500 mM NaCl and 20 mM NaH_2_PO_4_ solution at pH 7.0. For preparing ^15^N and/or ^13^C labeled proteins, a similar procedure was followed except cells were grown in isotopically labeled 1x M9 medium supplemented with ^15^NH_4_Cl (1 g/L), and ^13^C glucose (2 g/L). After purification, samples for NMR studies were dialyzed and buffer exchanged with 20 mM NaH_2_PO_4_ and 50 mM NaCl at pH 6.0 by centrifugation with 10,000-Da MWC membrane ultrafiltration (Millipore, Cork, Ireland). Samples used for X-ray crystallization, size-exclusion chromatography (SEC) coupled with multi-angle static light scattering (SEC-MALS) and circular dichroism (CD) experiments were further purified by SEC, superdex-75 (GE Healthcare Life Sciences, Uppsala, Sweden) with 20 mM Bis-Tris, 50 mM NaCl. Each fraction of the peak was analyzed on SDS-PAGE and concentrated by centrifugation (Amicon cell units Millipore). The purities of the purified proteins were validated by using SDS-PAGE, and their concentration was calculated by using a molar absorption coefficient of E^280^ = 9970 M^−1^ cm^-1^. Three HPLC-grade purified peptides, Myb3_52–59_, *Tv*CyP2_1–14_ (MLAFFATRVISAPK) and *Tv*CyP2_3–18_ (AFFATRVISAPKVTKK), used in this study were purchased from Yao-Hong Biotechnology (Taipei, Taiwan).

### 2.3. SEC-MALS Experiment

SEC-MALS analysis for molecular weight and oligomer state determination was performed as described [35]. Briefly, *Tv*CyP2 (1 mg/mL) and *Tv*CyP2-∆N (1 mg/mL) were used for SEC-MALS analysis. The column (Enrich Tm SEC. 70 10 × 300, Bio-Rad Laboratories, Santa Barbara, CA, USA) was used with a flow rate of 0.5 mL/min in the buffer system of 20 mM Bis-Tris, 50 mM NaCl at pH 6.0 and 25 °C. Detectors such as an ultraviolet-visible (UV) detector (QELS, Wyatt Technology, Santa Barbara, CA, USA), a static light-scattering detector (mini DAWN TREOS, Wyatt Technology, Santa Barbara, CA, USA), a quasi-elastic light-scattering detector (QELS, Wyatt Technology, Santa Barbara, CA, USA), and a refractive index detector (Optilab T-rEX, Wyatt Technology, Santa Barbara, CA, USA) all were aligned with the column. Bovine serum albumin (Sigma, A1900, Saint Louis, MO, USA) was used as a standard for calibration and optimization. The molecular weight was calculated by using ASTRA 6 with dn/dc value set to 0.185 mL g^-1^.

### 2.4. Crystallization Screening and X-ray Data Collection

Initial crystallization screening of *Tv*CyP2 involved using commercial crystallization screen kits (Hampton Research, Jena Bioscience, and Qiagen, Aliso Viejo, CA, USA), 96-well Intelli-plates (Art Robbins Instruments, Sunnyvale, CA, USA) and a Phoenix robot (Art Robbins Instruments, Sunnyvale, CA, USA) at 298 K. The sitting drop vapor diffusion method [38,39] was used to grow crystals. The 0.6–3 µm crystals of *Tv*CyP2 appeared within 5 days in the commercial kit conditions. After optimization, cylindrical-shaped crystals of *Tv*CyP2_apo1 were obtained at 288 K within 5–10 days under the condition of 0.1 M citric acid, 1.6 M ammonium sulfate at pH 5.0. To ensure that the determined X-ray structure was not purely due to crystal packing, three other kinds of crystals grown at different conditions were also used for X-ray study. For data collection, the final optimized crystals were cryoprotected in mother liquor supplemented with 10% glycerol and flash-frozen in liquid nitrogen at 100 K and mounted on an ADSC QUANTUM 315r detector in TLS beamline 13B1 or 13C1 at the National Synchrotron Radiation Research Center (Hsinchu, Taiwan). The protein crystal giving the best diffraction was finally placed for complete data collection with 180 frames, 300-m detector distance, and beam size 200 µm. All collected data were processed and scaled by using HKL2000 [40].

### 2.5. Refinement and Structure Determination

The X-ray structures of *Tv*CyP2 were determined by the molecular replacement method [41] with *Tv*CyP1 (PDB ID 5YBA) [35] used as a starting model with the Phaser-MR program [42]. This initial modeling was solved and improved by using COOT [43] and PHENIX [44] refinement. Refinement involved repeated cycles of conjugate-gradient energy minimization and temperature-factor refinement with the program Phenix.refine in the PHENIX package [45]. Amino-acid side chains and water molecules were fitted into 2Fo-Fc and Fo-Fc electron-density maps by using COOT. The model was evaluated using PROCHECK [46] and MOLPROBITY [47]. The atomic coordinates and structure factors for the four *Tv*CyP2 structures are deposited under RCSB PDB accession codes PDB: 6LXO, 6LXP, 6LXQ, and 6LXR, respectively.

### 2.6. NMR Spectroscopy

All NMR samples were prepared in buffer containing 20 mM NaH_2_PO_4,_ 50 mM NaCl at pH 6.0, with 10% D_2_O. All NMR spectra were acquired on Bruker AVANCE 600 and 800 MHz spectrometers equipped with a z-gradient TXI cryogenic probes (Bruker, Karlsruhe, Germany). Because we already published the backbone resonance assignment of *Tv*CyP2 by using 3D triple resonance experiments [48], we only performed 2D NMR experiments in this study, as described below. 2D ^1^H-^15^N HSQC titration experiments on both ^15^N-*Tv*CyP2 and ^15^N-*Tv*CyP2-∆N with *Tv*CyP2_3–18_ peptide were acquired at 800 MHz and 310 K. 2D ^1^H-TOCSY, ^1^H-COSY, and ^1^H-NOESY on the Myb3_52–59_ peptide were acquired at 600 MHz and 283 K to determine its proton resonance assignments. 2D ^1^H-ROESY was carried out with a mixing time of 300 ms at 283 K on the Myb3_52–59_ peptide (4000 μm) mixed with *Tv*CyP2 (20 μm) with the molar ratio of 200:1 for investigating peptidyl-prolyl isomerase activity. 2D ^1^H-TOCSY, ^1^H-COSY, and ^1^H-NOESY were acquired at 800 MHz and 283 K on 1.3 mM *Tv*CyP2_3–18_ peptide at different membrane mimetic conditions, such as 30% Trifluroethanol (TFE), 100 mM SDS and dodecylphosphocholine (DPC) micelles. All NMR spectra were processed by using Topspin 3.1 (Bruker) and analyzed by using NMRViewj [49]. The backbone NMR resonance assignment of *Tv*CyP2 has been deposited in BMRB under accession number 27033.

### 2.7. CD Spectroscopy

Far-UV CD spectra for *Tv*CyP2_3–18_ peptide (20 μm), *Tv*CyP1 (15 μm), *Tv*CyP2 (15 μm), *Tv*CyP2-∆N (15 μm) and *Tv*CyP2-∆N (15 μm) in complex with *Tv*CyP2_3–18_ peptide (1:2 molar ratio) and *Tv*CyP2_3–18_ (20 μm) in 30% TFE, SDS and DPC micelles were acquired by using a Chirascan^TM^ spectropolarimeter (Applied Photophysics, Surrey, UK) in 20 mM NaH_2_PO_4_ at pH 6.0 with a 1-mm path-length quartz cuvette at 298 K. All spectra were averaged over three scans and converted to the mean residue ellipticity, [*θ*]. For measuring melting temperature (Tm), the transition in the far-UV CD signal at 222 nm was monitored as a function of increasing temperature from 20 to 94 °C with a scan rate of 1 °C/min.

### 2.8. Immunofluorescence Assay (IFA)

*T. vaginalis* T1 cells [36] Cells were fixed in 4% paraformaldehyde in phosphate-buffered saline (PBS) for 15 min and permeated in 0.2% Triton X-100 in PBS for 15 min. The primary immunoreaction involved use of the mouse anti-HA antibody (100×) (HA-7, Sigma, Darmstadt, Germany); secondary immunoreactions involved FITC-conjugated antibody (200×) (Jackson Immuno Research, West Grove, PA, USA). Nuclei were stained with DAPI. Fluorescence signals were measured by confocal microscopy (LSM700, Zeiss, Jena, Germany). Cell morphology was imaged by phase-contrast microscopy.

### 2.9. Immunoprecipitation (IP)

*T. vaginalis* T1 cells [36] were used to identify the complex formation of *Tv*CyP2 with its interacting counterparts by immunoprecipitation. First, 7.5 × 10^7^ cells were solubilized with lysis buffer (1% Triton X-100, 1x Protease inhibitor cocktail, and 200 μg/mL TLCK in PBS). The protein extracts were 5-fold diluted in PBS and then each incubated with 20 μL agarose beads conjugated with anti-HA antibody (Sigma) on gentle rotation at 4 °C overnight. The beads recovered with centrifugation at 1000× *g* were washed three times by PBS containing 0.1% Triton X-100. The immunoprecipitants were boiled in 1× SDS sample buffer for subsequent Western Blotting detection. For each assay, the protein lysates from non-transgenic parasites serve as the control for immunoprecipitation.

### 2.10. Western Blot Analysis

Protein samples were purified as described [36] and separated by SDS-PAGE. The SDS-separated protein was stained with Coomassie blue or transferred to polyvinylidene difluoride membranes (PVDF) for Western blotting using the rabbit anti-acetyl histone H3K9 (3000×) (Upstate, NY, USA), a mouse monoclonal anti-α-tubulin antibody (10,000×) (DMIA, Sigma), and a mouse monoclonal anti-HA antibody (5000×), rat anti-*Tv*CyP1 (5000×) [50], rat anti-*Tv*CyP2 (1000×) [51], rabbit anti-HdHSP70 (10,000×) [52], and rabbit anti-*Tv*Bip (10,000×) [52]. Signals were detected by enhanced chemiluminescence (ECL), as described by the supplier (Thermo Scientific, Waltham, MA, USA). The relative intensities of the signal were quantified and analyzed using MetaMorph (Molecular Devices, San Jose, CA, USA).

## 3. Results

### 3.1. TvCyP2 Forming a Monomer and Possessing PPIase Activity

**Figure 2:**
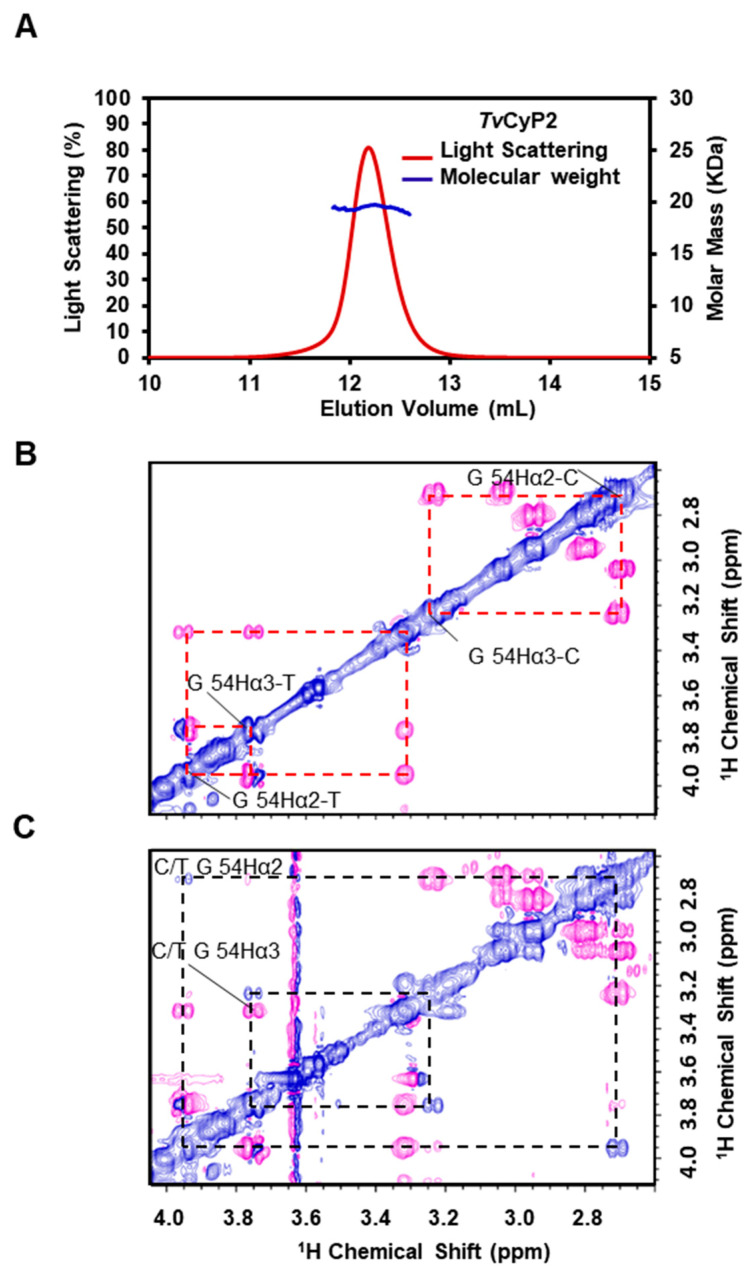
(**A**) Size-exclusion chromatography coupled with multi-angle static light scattering (SEC-MALS) analysis showing that *Tv*CyP2 forms a monomer in solution with molecular weight 20.91 kDa. (**B**) The selected region of 2D ROESY spectrum on Myb3_52–59_ peptide acquired with mixing time 300 ms at 283 K is shown, with the positive and negative cross-peaks in blue and pink, respectively. Because *cis* and *trans* conformers of Myb3_52–59_ peptide are slowly exchanged, the cross peaks of C-G54 CH_α2_/C-G54 CH_α3_ and T-G54 CH_α2_/T-G54 CH_α3_ were simultaneously observed in the spectrum. (**C**) When adding *Tv*CyP2, the exchange rate between the *cis* and *trans* conformations is greatly increased, so that cross peaks due to fast exchange appeared, such as C-G54 CH_α2_/T-G54 CH_α2_ and C-G54 CH_α3_/T-G54 CH_α3_ cross peaks shown in blue.

*Tv*CyP1 is known to form a dimer in both crystal and solution states [35]. With the SEC-MALS experiment, the molecular weight of *Tv*CyP2 was measured at ~20.01 kDa in a solution similar to theoretical molecular weight 20.947 kDa (Figure 2A), *Tv*CyP2 forms a monomer, which agrees well with most of the single-domain CyPs but not *Tv*CyP1. The PPIase activity of *Tv*CyP2 was investigated by using the Myb3_52–59_ peptide that contains a Gly-Pro bond as a substrate in 2D ROESY NMR experiments. In a ROESY spectrum, the sign of the cross-peak due to chemical exchange is the same as that of the diagonal peak, whereas the cross-peaks between neighboring hydrogens display an opposite sign. Two sets of NMR resonances were detected in the Myb3_52–59_ peptide in the absence of *Tv*CyP2, so the *cis*/*trans* interconversion in peptide alone is slow on the NMR time scale (Figure 2B). When adding *Tv*CyP2, the *cis/trans* isomerization rate was enhanced so that new cross-peaks due to chemical exchange, such as cis-G54 C_α_H/trans-G54 C_α_H, were seen (Figure 2C), which confirms that *Tv*CyP2 is a catalytic enzyme and possesses PPIase activity.

### 3.2. X-ray Crystal and NMR Solution Structures of TvCyP2

**Figure 3:**
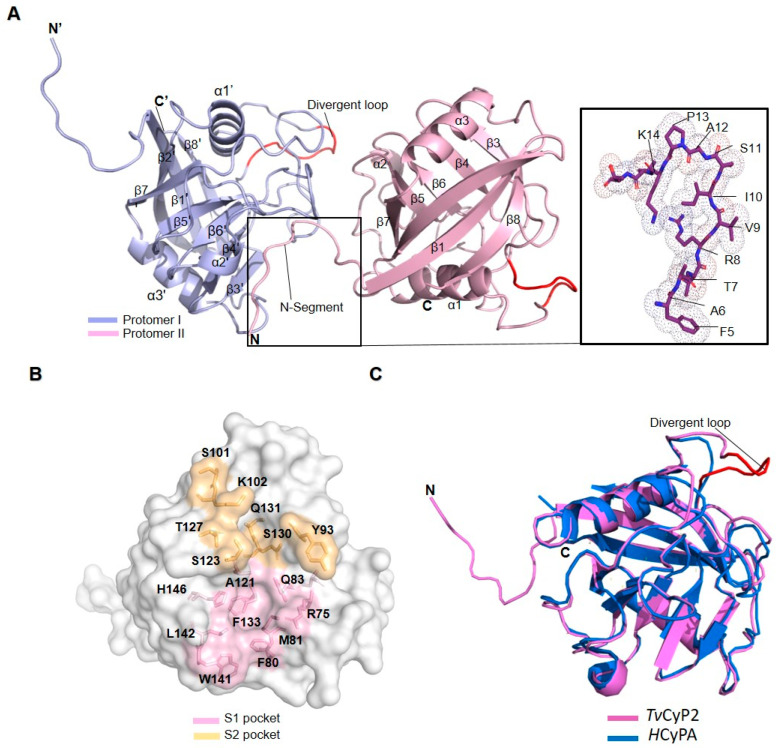
(**A**) The X-ray crystal structure of *Tv*CyP2 has a typical canonical cyclophilin fold and contains 3 α-helices and 8 β-strand secondary elements, with the divergent loop from residues 61–67 in red. The N-terminal segment is associated with a neighboring *Tv*CyP2, which is highlighted in the box with the conformation of the N-terminal F5 to K14 shown in the right box. (**B**) Surface structure of *Tv*CyP2 showed the distribution of both active-site and S2 pocket residues in pink and orange, respectively. (**C**) Structural comparison by superimposing the *Tv*CyP2 structure in magenta with *h*CypA in blue, with the divergent loop shown in red.

Using the ARI crystallization robot, we performed crystal screenings of *Tv*CyP2 under various conditions and found that four needle-shaped crystals gave good X-ray diffraction data, all belonging to the space group p2(1)2(1)2(1). These four X-ray structures were solved by molecular replacement with the *Tv*CyP1 structure (PDB 5YB9) used as a search model, and the best structure had 1.89 Å resolution with R (work) = 14.02 and R (free) = 17.93. The Ramachandran plot showed that 96.0% of residues lay within the most favored regions, with no residues in the generously disallowed region. The refinement details of all four X-ray structures are in Table 1. The secondary structures of *Tv*CyP2 contain 8 β-strands and 3 α-helices, β1: residues 16–24, β2: 29–36, β3: 74–77, β4: 81–83, β5: 116–120, β6: 131–135, β7: 148–154, β8: 175–184, α1: 43–53, α2: 140–142 and α3: 157–166 arranged in order of β1–β2–α1–β3–β4–β5–β6–β6–α2–β7–α3–β8 (Figure 3A). The distribution of the conserved active site residues and the variable S2 pocket residues are labelled in surface structure (Figure 3B), and the structural alignment with *h*CypA showed that *Tv*CyP2 has a conserved divergent loop, residues 61–67 (KLGKPLH), located just above the active site pocket [53] (Figure 3C).

**Figure 4:**
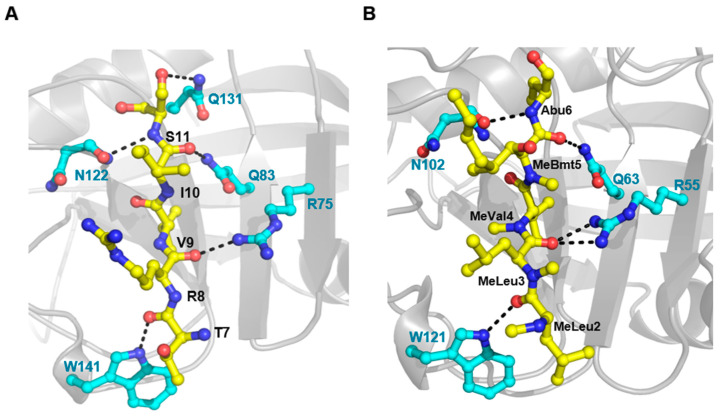
(**A**) The interactions between N-terminal segment and active site residues in *Tv*CyP2 contain 4 H-bonds shown by black dotted lines. (**B**) In comparison, 5 H-bonds are observed for the interactions between CsA and active site residues of *h*CyPA. Both Figures were generated by using PyMOL.

In all four crystal structures (Figure S1), except for forming a typical canonical cyclophilin fold, the N-terminal segment contacted the active site of a neighboring molecule (Figure 3A). A detailed analysis showed that these interactions include H-bonds formed by the N-terminal T7, R8, I10, and S11 with active site W141, R75, Q83, and N122 residues, respectively, and also by N-terminal S11 with Q131 of the S2 pocket (Figure 4A). In addition, some hydrophobic contacts were observed: N-terminal A6 with active site F80, N-terminal V9 with active site M81, A121, F133, L142, and H146, and N-terminal P13 with S2 pocket Y93. Because all known CyP structures lack this type of interaction, these particular interactions are considered interesting in CyPs. In comparing the *h*CyPA–CsA complex structure, the corresponding active-site residues in *h*CyPA, R55, Q63, N102, and W121 form H-bonds with CsA (Figure 4B), and F60, M61, A103, F113, L122, and H126 have hydrophobic contacts with CsA. Because both the N-terminal segment and CsA have similar interactions when binding to cyclophilin, this finding let us assume that the N-terminal segment behaves as a substrate like CsA in the *h*CyPA–CsA complex.

We previously published the secondary structure of *Tv*CyP2 in solution based on the chemical shift index [48], which is mostly in good agreement with the X-ray structure. From the X-ray structure, we could well explain the effect causing the unusual chemical shift on some specific residues. For example, amide protons of K44, T45, and D86 are deshielded because of H-bond formations. S107 and K69 are shielded because of the ring current effects from various Phe and Tyr residues in their vicinity. Also, the unusual downfield chemical shift of K61 is affected by a strong H-bond formation with E96, and because of this H-bond, the divergent loop can be kept in a particular conformation. Accordingly, the solution structure and X-ray structure of *Tv*CyP2 should be highly similar. However, whether the interaction between the N-terminal segment and the active site observed in the X-ray structure occurs in solution remained unknown.

### 3.3. Self-Association of the N-Terminal Segment also Observed in Solution

**Figure 5:**
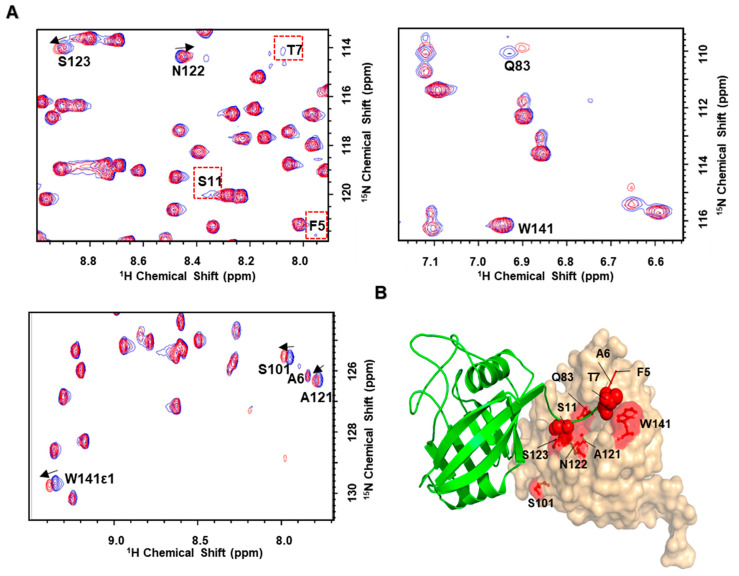
(**A**) Three particular regions of the superimposed 2D ^1^H-^15^N HSQC spectra of ^15^N-labelled *Tv*Cyp2 in the absence and presence of unlabeled *Tv*CyP2_3–18_. The cross peaks that show chemical shift perturbation or line width change between the two spectra are labelled. (**B**) Residues with shift perturbation or line width change are mapped onto the *Tv*CyP2 structure with residues from the active site shown in red sticks (red) and from the N-terminal segment in red spheres.

To provide a definitive answer, we carried out more NMR studies. Initially, *Tv*CyP2_1–14_ was synthesized for further NMR study. Unfortunately, this peptide is too hydrophobic to be dissolved in the aqueous buffer, so another 16-residue peptide, *Tv*CyP2_3–18_, was synthesized and used for NMR study because it is highly soluble and gives good NMR data. Comparison of 2D ^1^H-^15^N HSQC spectra for ^15^N-labelled *Tv*CyP2 without and with *Tv*CyP2_3–18_ (molar ratio 1:5) showed that active-site residues Q83, A121, N122, and W141 and S2 pocket residues S101, S123 and S130 have significant chemical shift perturbations, and also N-terminal F5, A6, T7, and S11 have the line width broadened or chemical shift perturbations (Figure 5 and Figure S2A). These perturbed residues were mapped onto the crystal structure, which clearly shows their distribution (Figure 5B). Therefore, in solution, the N-terminal segment should also interact with the active site but with weak binding affinity, and when *Tv*CyP2_3–18_ is added, it can compete and replace the N-terminal segment to interact with the active site. Once the N-terminal peptide is replaced by *Tv*CyP2_3–18_, chemical environments on those interacting residues are changed, hence causing chemical shift changes or line-width broadenings. Additionally, the corresponding active-site residues on *Tv*CyP1, such as Q71, S107, A109, N110, A111, S118, and W129, were perturbed when *Tv*CyP1 was titrated with *Tv*CyP2_3–18_ (Figure S2B), which illustrates that the N-terminal segment of *Tv*CyP2 interacts with its own active site and also with that of other CyPs, such as *Tv*CyP1.

### 3.4. Thermostability and Structure of TvCyP2 without N-Terminal Segment (TvCyP2-∆N)

**Figure 6:**
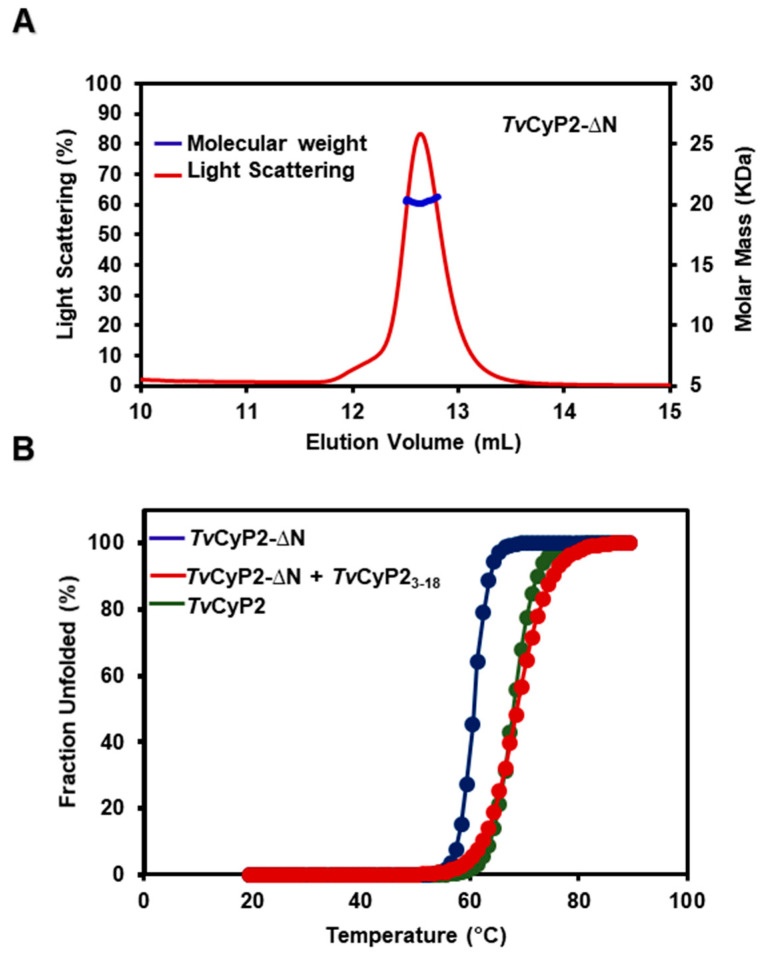
(**A**) SEC-MALS experiment showing that *Tv*CyP2-∆N forms a monomer in solution with molecular weight 19.5 kDa. (**B**) Thermal unfolding CD spectra for *Tv*CyP2 and *Tv*CyP2-∆N and *Tv*CyP2-∆N mixed with *Tv*CyP2_3–18_, shown in green, blue and red, respectively, from 20 to 90 °C at wavelength 222 nm. *Tv*CyP2 with T_m_ 70 °C was more stable than *Tv*CyP2-∆N with T_m_ 61 °C. For *Tv*CyP2-∆N mixed with *Tv*CyP2_3–18_, the melting temperature is increased, nearly the same as that of *Tv*CyP2.

To further gain insights into the structural and functional roles of the N-terminal segment, we performed some experiments with *Tv*CyP2-∆N. The SEC-MALS experiment clearly showed that *Tv*CyP2-∆N forms a monomer in solution (Figure 6A). CD melting temperature study showed *Tv*CyP2 is more thermally stable than *Tv*CyP2-∆N (Figure 6B) and even *Tv*Cyp1 (Figure S3). When *Tv*CyP2-∆N was mixed with the *Tv*CyP2_3–18_ peptide, the melting temperature was nearly the same as for *Tv*CyP2 (Figure 6B). This finding indicated that *Tv*CyP2_3–18_ peptide can act like the N-terminal segment to associate with the active site of *Tv*CyP2-∆N so that the mixture of *Tv*CyP2-∆N with *Tv*CyP2_3–18_ behaves like *Tv*CyP2, as do their melting temperatures.

The superimposed 2D ^1^H-^15^N HSQC spectra of *Tv*CyP2-∆N and *Tv*CyP2 showed that most cross peaks are identical and the cross-peaks missing on *Tv*CyP2-∆N are due to lack of the N-terminal segment. Residues with significant shift perturbations, such as S101 A121, N122, S123, S130, W141, and H146, are mostly from the active site and S2 pocket sites, and few are from the N-terminal segment or its nearby residues (Figure S4A,B). Therefore, with the N-terminal segment excluded, the tertiary structures of both *Tv*CyP2-∆N and *Tv*CyP2 are greatly alike.

### 3.5. N-Terminal Segment Affects the Subcellular Localization and Formation of Differential Complexes in Cellular Processes

**Figure 7:**
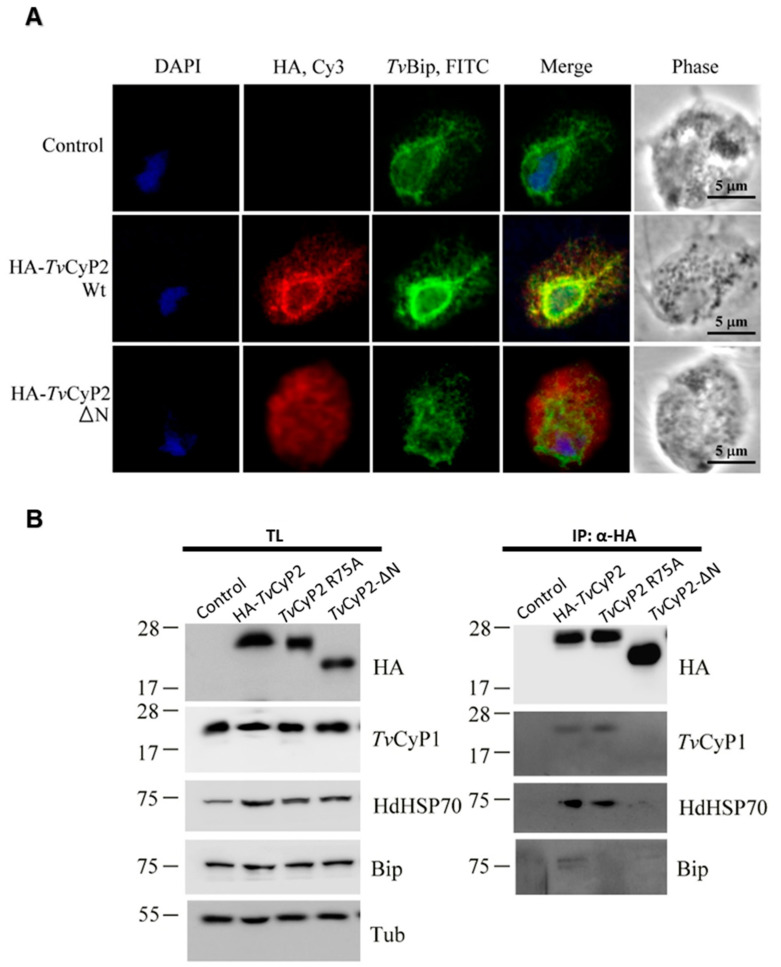
(**A**) Subcellular localization of hemagglutinin cyclophilin 2 (HA-CyP2) and HA-CyP2-ΔN in *Trichomonas vaginalis* were determined by immunofluorescence assay (IFA). The two transgenic and non-transgenic parasites (control) were double-stained by the mouse anti-HA and rabbit anti-*Tv*Bip antibodies, followed by reacting with Cy3- and FITC-conjugated secondary antibodies. The Cy3 and FITC signals indicated the HA-CyP2 and *Tv*Bip localization, respectively. Nuclei were stained by DAPI. Fluorescence signal was observed by a confocal microscope. (**B**) Total lysates (TL) extracted from transgenic and non-transgenic parasites (control) were examined by Western blotting (left panel), or further immunoprecipitated (IP) by the agarose beads conjugated with anti-HA antibody for subsequent Western blot detection (right panel). The detected proteins by Western blot were indicated at the right side, and molecular weights were labeled at the left side of each panel.

Because the N-terminal segment of *Tv*CyP2 is highly hydrophobic and has a unique structural feature, we wondered whether the N-terminal segment plays any biological function. For this purpose, we established transgenic cells overexpressing HA-*Tv*CyP2 and HA-*Tv*CyP2-∆N. We performed IFA experiments with anti-HA antibody to observe the localization of HA-*Tv*CyP2 and HA-*Tv*CyP2-∆N. The FITC signals showed HA-*Tv*CyP2 mainly surrounding the nucleus and extending to the cytosol. As shown earlier [36], wild-type HA-*Tv*CyP2 proteins were retained in the ER and membrane compartments of downstream transport networks. *Tv*CyP2 was mainly localized around the nucleus, with FITC signals surrounding the nucleus. In comparison, with *Tv*CyP2-∆N, the ER signal was eliminated and the cytosolic signal was enhanced (Figure 7A). This finding suggests that the N-terminal segment plays a vital role in regulating the subcellular localization of *Tv*CyP2.

In our previous study, we have shown that the protein complexes immunoprecipitated from lysates of *Tv*CyP2 and *Tv*CyP2 R75A overexpressing cells have shown effects on the expression of other proteins [36]. Here we have compared the same with *Tv*CyP2-∆N. As the N-terminal segment of *Tv*CyP2 is crucial in the functional and structural aspect of protein; therefore, it was interesting to see the effects of N-terminal segment deletion in the expression of other enzymes. Also, the enzymatic activity and integrity of the catalytic domain are essential for interacting with other binding partners; therefore, we used mutant R75A and *Tv*CyP2-∆N in this study. To examine its role in the expression of other proteins, we examined cell lysates from control and transgenic cells overexpressing HA-*Tv*CyP2, *Tv*CyP2-∆N and R75A by Western blotting (Figure 7B, left panel). Similar amounts of HA-*Tv*CyP2, *Tv*CyP2-∆N and R75A were detected in samples from transgenic cell lines. The overexpression of *Tv*CyP2 and its derived mutants exert a little effect on the overall expression of *Tv*CyP1 and *Tv*Bip samples. Interestingly, HdHSP70 was detected to a substantially higher level in samples from transgenic cells line than control, implying a possible regulation of *Tv*CyP2 in the expression of HdHSP70. The N-terminal segment showed little effect on the expression of HDHSP70 as there is no significant difference in the expression of HdHSP70 by *Tv*CyP2-∆N with compared to HA-*Tv*CyP2 and *Tv*CyP2(R75A).

Furthermore, we examined the role of the N-terminal segment in the complex formation of *Tv*CyP2 with its interacting counterparts. The same samples from total lysates were immunoprecipitated with an anti-HA antibody for Western blotting (Figure 7B, right panel). *Tv*CyP2-∆N aborted the complex formation of *Tv*CyP2 with *Tv*CyP1, HdHSP70 and *Tv*Bip as compared with the mutant *Tv*CyP2(R75A). These observations suggest that the deletion of the N-terminal segment seems to affect differential complex formation.

### 3.6. N-Terminal Segment in Membrane Mimicking Environments Forming an α-Helical Structure

**Figure 8:**
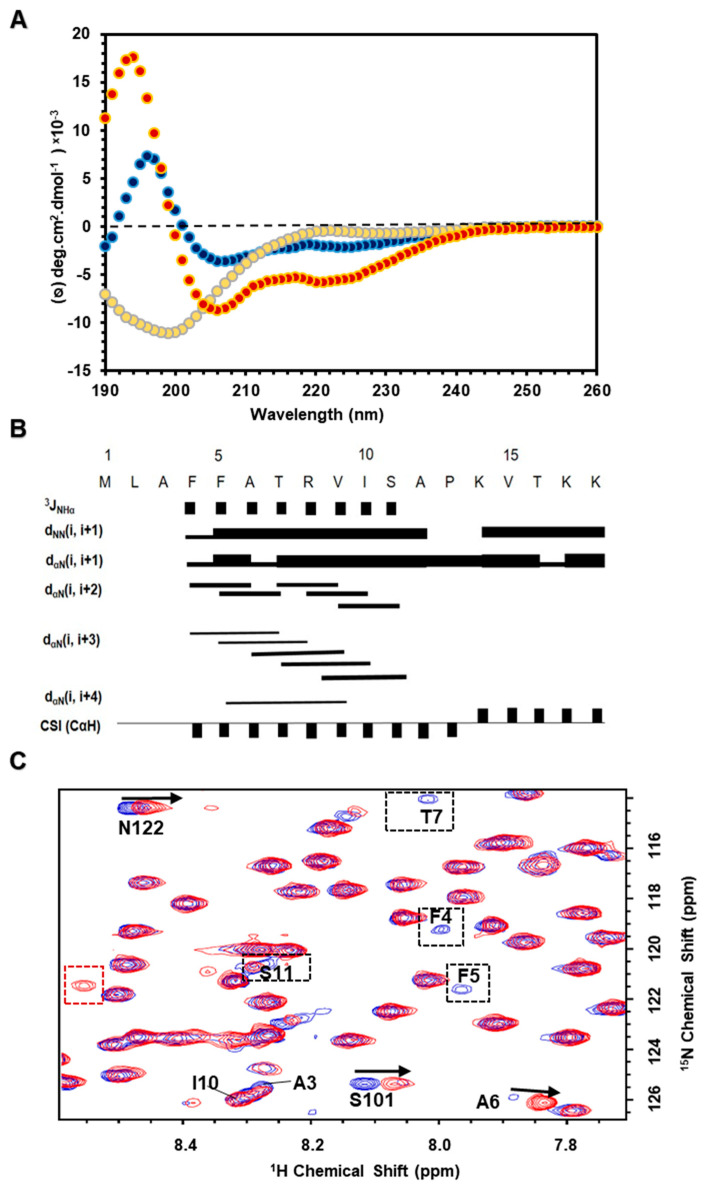
(**A**) Comparison of CD spectra for *Tv*CyP2_3–18_ peptide in aqueous buffer (yellow), 30% Trifluoroethanol (TFE) (red) and sodium dodecyl sulfate (SDS) micelles (blue) shows that *Tv*CyP2_3–18_ in TFE and SDS micelles forms an α-helical structure because two minimum absorbances at 208 and 222 nm are observed in their spectra. (**B**) Summary of the structural statistics of *Tv*CyP2_3–18_ peptide in 30% TFE showing the primary sequence, ^3^J_NHα_ coupling constants, intensities of particular NOE connectivities, and C_α_H chemical shift index. ^3^J_NHα_ coupling constants < 6 Hz are indicated by filled squares. Bar thickness indicates the intensity of Nuclear overhauser effect (NOE) connectivity, with a thicker bar representing stronger NOEs. The negative bars in the chemical shift index indicate upfield shifts of > 0.1 ppm of the C_α_H as compared with the expected random-coil C_α_H chemical shift. The positive bars indicate downfield shifts of >0.1 ppm of the C^α^H as compared with the expected random-coil C_α_H value. (**C**) A partial region of the 2D ^1^H-^15^N HSQC spectra of *Tv*Cyp2 in aqueous solution overlapped with that in DPC micelles (Figure S5A). Residues showing chemical shift perturbation or line width broadening are labelled.

As described above, in vivo studies, it is possible that N-terminal segment is involved in ER targeting or ER retention. Thus, the N-terminal segment may interact with the membrane. Accordingly, we used a biophysical study of the *Tv*CyP2_3–18_ peptide in membrane mimetic environments, such as TFE organic solvent, SDS, and DPC micelles. CD experiments showed that *Tv*CyP2_3–18_ was unstructured in aqueous buffer and contained an α-helical structure in membrane mimetic conditions (Figure 8A). NMR spectra of *Tv*CyP2_3–18_ peptide in 30% TFE at pH 6.0 and 283 K were well dispersed, so NMR resonance assignments were accomplished in a short time. From the C_α_H chemical shift index (CSI) and the Nuclear overhauser effect (NOE) connectivity, especially medium-range NOEs characteristic of an α-helical structure, the secondary structure of *Tv*CyP2_3–18_ peptide was derived to contain an α-helix from residues 4–12 (Figure 8B). Moreover, we acquired 2D ^1^H-^15^N HSQC spectra of *Tv*CyP2 and *Tv*CyP2-∆N in the absence and presence of DPC micelles. The superimposed spectra on *Tv*CyP2 showed that NMR resonance line widths of N-terminal F4, F5, T7, and S11 were broadened, and cross peaks of active site S101 and N122 residues were significantly perturbed (Figure 8 and Figure S5A). By contrast, the superimposed spectra on *Tv*CyP2-∆N showed that all cross-peaks were nearly identical, that is, no interaction between *Tv*CyP2-∆N and DPC micelles. These findings verified that the N-terminal segment is required for interacting with DPC micelles (Figure S5B).

## 4. Discussion

Previously, we reported the novel structural characteristics of *Tv*CyP1 and its complex with Myb1_104–111_ peptide [35]. In comparing both *Tv*CyP1 and *Tv*CyP2 structures, we found two major structural differences. First, *Tv*CyP1 forms a dimer, but *Tv*CyP2 is monomeric. Second, the N-terminal segment of *Tv*CyP2, lacking in *Tv*CyP1, possesses two unique structural features. It associates with the active site of a neighboring *Tv*CyP2 and also has a tendency to interact with the membrane. These structural differences may cause different mechanisms in regulating Myb1 and Myb3 protein trafficking between *Tv*CyP1 and *Tv*CyP2. Because the N-terminal segment of *Tv*CyP2 exhibits such unusual interactions with the active-site residues, one may wonder whether this interaction is real or purely due to crystal packing. The four X-ray crystal structures of *Tv*CyP2 under variable conditions were solved, all showing that the N-terminal segment interacts with the active site, so this remarkable interaction is real. The NMR chemical shift perturbation study further verified that this unique interaction also existed in solution. As shown by CD experiment, the differences in melting temperatures, with and without the N-terminal segment indicate that the N-terminus is playing a role in protein stability to some extent, which implies that the self-association likely exists even in a lower concentration.

We investigated all known CyP structures and found that the N-termini of the two human spliceosomal cyclophilins, *h*PPWD1 [54] and *h*CyPH [55], show interactions similar to *Tv*CyP2. In *h*PPWD1, the crystal structure of the C-terminal isomerase domain from residues 483–646 was solved: the N-terminal 7 residues, Glu483–Arg489 (QAEGP_487_KR), bound to the active site of an adjacent molecule. Among these 7 residues, Pro487 is the key residue that interacts with the active site by the formation of H-bond and hydrophobic interactions. In *h*CyPH, N-terminal P8 is the primary residue interacting with the active-site R67 and H138 residues of a neighboring molecule by the formation of water-mediated H-bonds. Thus, in both *h*PPWD1 and *h*CyPH CyPs, the N-terminal proline residue plays a key role in interacting with active-site residues of an adjacent molecule, which is expected because both *h*PPWD1 and *h*CyPH have PPIase activities that in theory tend to interact with proline residue [56]. By contrast, in *Tv*CyP2, the N-terminal P13 showed a hydrophobic contact with only Tyr93 of the S2 pocket but not with active-site residues. As a result, the interaction of the N-terminal segment with the active site in *Tv*CyP2 is unique and novel because it is not mainly mediated by a proline residue.

Comparison of the interactions among N-terminal segment with *Tv*CyP2, Myb1_104–111_ with *Tv*CyP1, and CsA with *h*CyPA [35,57] is displayed in Figure S6 and Table S1. The same active-site atoms, including the side chain NH_2_ of R75, side chain NH_2_ of Q83, backbone oxygen of N122 and side chain NH_ε1_ of Trp141 in *Tv*CyP2 and the corresponding atoms in both *Tv*CyP1 and *h*CyPA all formed an H-bond. Except for these conserved H-bonds, the side chain NH_2_ of active-site R55 in the *h*CyPA–CsA complex showed one more H-bond. The formation of this extra H-bond at an active site may explain why the *h*CyPA–CsA complex exhibits a much stronger binding affinity (nM) than the other two complexes (μM) [35,58]. Perhaps the macrolide structure and bulky side-chain groups in CsA play other roles in enhancing the binding affinity. Because CsA is able to tightly bind with *h*CyPA and other CyPs, it has long been considered a potent inhibitor of CyPs. Whether the N-terminal segment could be a good inhibitor like CsA to block the catalytic activity of CyPs deserves further investigation. NMR shift perturbation experiments showed that the active-site residues were only slightly perturbed when *Tv*CyP1 mixed with *Tv*CyP2_3–18_, which indicates that the binding affinity in the *Tv*CyP1–*Tv*CyP2_3–18_ complex is weak, so *Tv*CyP2_3–18_ alone is not a good inhibitor yet. Accordingly, the peptide needs to be further engineered and modified as a potent inhibitor.

IFA assay revealed that *Tv*CyP2 is highly concentrated around the nuclear membrane and ER, but *Tv*CyP2-∆N diffuses predominantly into the cytoplasm and ER. Moreover, immunoprecipitation has shown that the N-terminal segment seems to mediate protein-protein interactions. The removal of differential complex formation due to deletion of the N-terminal segment indicates that *Tv*CyP2 interactions with *Tv*CyP1, HdHSP70 and *Tv*Bip are mediated by the N-terminal segment. However, it needs further validation to understand why the N-terminal segment would induce complex formations with its interacting counterparts. NMR and CD experiments demonstrated that *Tv*CyP2_3–18_ likely have the potential to associate with the membrane because its conformation is induced to be an α-helix in membrane mimetic conditions. There is one PPIase, peptidyl peptidyl–prolyl isomerase d (PPid), that anchors the membrane via the N-terminal segment, and its catalytic region faces the cytoplasm [59]. However, it remains elusive for *Tv*CyP2 as the in vivo topology of *Tv*CyP2 is not yet fully known. Physiologically, *Tv*CyP1, *Tv*CyP2, and Myb3 possibly co-exist within the ER, Golgi and hydrogenosomes and interact with each other spatially and temporally, thereby fulfilling the functional role in *T. vaginalis* [36]. *Tv*CyP2 might play a role in trafficking *Tv*CyP1, Myb3, or other proteins by anchoring to the membrane and providing its accessible catalytic pocket for substrate binding or modulation of proline switches. By anchoring to the membrane, it may also help the newly synthesized protein to reach its final conformation and destination by using its active site as a chaperone. The extended sequences at the N-terminal segment in *Tv*CyP2 may be crucial to import the protein in organelles [60]. It is well known that signal peptide is composed of three regions: N-region containing positive charge, H-region containing hydrophobic core and C-region having cleavage site [61]. However, N-terminal segment only contains a consensus H-region, and lack of both N- and C-regions. Moreover, the N-terminal tagged HA-CyP2 was detected by Western blot as a single band in total lysates as well as in various membrane fractions either by anti-*Tv*CyP2 or anti-HA antibody [36], suggesting that cleavage of the N-terminal region if exists is less likely a major event. Likewise, many other hydrogenosomal proteins, including *Tv*CyP1 and Myb3, are also devoid of a signal peptide sequence targeting from the cytosol to the hydrogenosome [62,63]. Therefore, *Tv*CyP2 may function differently from the conventional targeting pre-sequences, so it harbors a novel mechanism for trafficking proteins.

Our current findings from X-ray structure and NMR analysis suggest a possibility that *Tv*CyP2 might be in an inactive state in the cytoplasm, and its active site occupied with the N-terminal segment. By contrast, in the ER, *Tv*CyP2 co-exists with Myb3 and *Tv*CyP1, where *Tv*CyP2, may play an important role for *cis/trans* conformational switches or binding with *Tv*CyP1 and Myb3 through its active site. However, it still needs further research and analysis to clarify the secretory pathway of proteins to understand the biological perspective.

## 5. Conclusions

Translocation of transcription factors to the nucleus is a decisive step for cytoadherence in trichomoniasis. *Tv*CyP2 is an important member of the endomembrane protein trafficking pathway, so characterizing the structural and functional features of the N-terminal segment, responsible for enzyme localization and potential role in association with the membrane, opens an avenue for understanding how *Tv*CyP1 and *Tv*CyP2 mediate nuclear transport for the Myb transcription factors in the disease progression. The self-association of the N-terminal segment with active site residues gives an additional clue to the development of a specific potent inhibitor for CyPs, which may be further applied in drug development for combatting the disease caused by the pathogenic parasite.

## Figures and Tables

**Table 1:** Data collection and refinement statistics of X-ray structures of *Tv*CyP2.

**Data Collection**				
**Crystal**	*Tv*CyP2_apo1	*Tv*CyP2_apo2	*Tv*CyP2_apo3	*Tv*CyP2_apo4
**Space group**	*P*2_1_2_1_2_1_	*P*2_1_2_1_2_1_	*P*2_1_2_1_2_1_	*P*2_1_2_1_2_1_
**Cell dimensions**				
***a, b, c* (Å)**	50.8, 56.1, 59.8	53.8, 54.2, 59.8	54.3, 54.6, 59.9	52.0, 54.5, 59.6
***α,*** ***β,*** ***γ* (°)**	90.0, 90.0, 90.0	90.0, 90.0, 90.0	90.0, 90.0, 90.0	90.0, 90.0, 90.0
**Resolution (Å)**	28.09–1.89 (1.95–1.89)	27.13–2.35 (2.44–2.35)	27.15–1.85 (1.92–1.85)	26.17–2.56 (2.65–2.56)
***R_merge_*^a,b^**	0.039 (0.135)	0.070 (0.327)	0.055 (0.350)	0.077 (0.405)
***I/*** ***σ (I)*^a^**	33.8 (10.6)	16.8 (2.8)	23.9 (3.1)	18.5 (3.2)
**Completeness (%) ^a^**	98.2 (90.9)	91.4 (91.8)	99.7 (97.8)	99.9 (100.0)
**Redundancy ^a^**	4.7 (4.7)	4.5 (3.5)	4.6 (4.3)	6.6 (5.8)
**CC_1/2_^a,c^**	0.993 (0.979)	0.970 (0.919)	0.981 (0.924)	0.978 (0.918)
CC *	0.998 (0.995)	0.992 (0.979)	0.995 (0.980)	0.994 (0.978)
Refinement				
**Resolution (Å)**	28.09–1.89	27.13–2.35	27.15–1.85	26.17–2.56
**No. of reflections**	14,047	6685	14941	5340
***R_work_/R_free_*^d^**	0.1394/0.1785	0.1551/0.2254	0.1463/0.1821	0.1677/0.2308
**No. of atoms**				
**Protein**	1350	1363	1374	1366
**Ligand/ion**	42		61	
**Water**	196	26	106	22
***B*-factor**				
**Protein**	18.91	32.78	21.67	32.85
**Ligand/ion**	63.93		64.08	
**Water**	32.60	35.07	35.41	31.99
**R.m.s. deviation**				
**Bond lengths (Å)**	0.007	0.008	0.007	0.008
**Bond angles (°)**	1.19	1.28	1.26	1.25
**Ramachandran plot ^e^**				
**Favoured (%)**	96.0	94.4	96.6	95.5
**Allowed (%)**	3.9	5.0	3.3	4.4
**Outliers (%)**	0	0.5	0	0

^a^ Values within parentheses are for highest-resolution shell. ^b^ *R*_merge_ = Σ_h_Σ_i_|*I_h_*_,*i*_ − *I_h_*|/Σ_h_Σ_i_*I_h_*_,*i*_, where *I_h_* is the mean intensity of the *i* observations of symmetry related of h. reflections. C_1/2_ is a percentage correlation between intensities from random half-datasets. ^c^ CC_ano_ is a percentage correlation between random half-datasets of anomalous intensity differences. ^d^ *R*_work_/*R*_free_ = Σ|*F_obs_* − *F_calc_*|/Σ*F_obs_*, where *F_calc_* is the calculated protein structure factor from the atomic model (*R*_free_ was calculated with 5% of the reflections selected). ^e^ Percentage of residues in most favored/additionally allowed/generously allowed/disallowed regions of Ramanchandran plot, according to PROCHECK.

## Supplementary Materials

The following are available online at https://www.mdpi.com/2218-273X/10/9/1239/s1, Table S1: Comparison of interactions among N-terminal segment with *Tv*CyP2, Myb1104–111 peptide with *Tv*CyP1 (PDB ID 5YBA) and CsA with *h*CyPA (PDB ID 1CWL) according to the X-ray structural data, Figure S1: Structural superimposition of four X-ray structures, *Tv*CyP2_apo1 (cyan), *Tv*CyP2_apo2 (pink) *Tv*CyP2_apo3 (red) and *Tv*CyP2_apo4 (blue), determined under different conditions, Figure S2: (A) 2D 1H-15N HSQC spectra for TvCyp2 in blue overlapped with that for *Tv*Cyp2 titrated with unlabeled *Tv*CyP23-18 (1:5 molar ratio) shown in red. (B) 2D 1H-15N HSQC spectra for *Tv*Cyp1 in blue overlapped with that for *Tv*Cyp1 titrated with unlabeled *Tv*CyP23-18 (1:5 molar ratio) shown in red. Cross peaks that showed chemical shift perturbation or line width broadening upon adding the TvCyP23-18 peptide are labelled, Figure S3: Thermal unfolding CD spectra of *Tv*CyP2 and TvCyP1 shown in red and blue, respectively, from 20 to 90 °C at wavelength 222 nm, Figure S4: (A) The superimposed 2D 1H-15N HSQC spectra between *Tv*Cyp2 in blue and *Tv*Cyp2-∆N in red. Residues showing chemical shift perturbation between the two spectra and disappeared in *Tv*Cyp2-∆N due to the lack of N-terminal segment are annotated. (B) Residues with shift perturbations are mapped onto the structure, showing they are close to the N-terminal segment (blue) or in the active-site pocket and its nearby region (red), Figure S5: (A) The overlapped 2D 1H-15N HSQC spectra of *Tv*CyP2 in aqueous solution (blue) and in the presence of DPC micelles (red). Cross peaks with shift perturbation, line width changing, and disappeared are labelled. (B) The overlapped 2D 1H-15N HSQC spectra of *Tv*CyP2-∆N in aqueous solution (blue) and in the presence of DPC micelles (red), Figure S6: (A) H-bond and hydrophobic interactions observed between N-terminal segment and a neighboring *Tv*CyP2. (B) H-bond and hydrophobic interactions observed in *h*CyPA–CsA complex. (C) H-bond and hydrophobic interactions observed in *Tv*CyP1–Myb1105-110 complex.
