# Supplementary material for: N-Terminal Segment of TvCyP2 Cyclophilin from Trichomonas vaginalis Is Involved in Self-Association, Membrane Interaction, and Subcellular Localization
Source: Biomolecules. 2020 Aug 26;10(9):1239. doi: 10.3390/biom10091239 (PMC7563477; doi:10.3390/biom10091239)
Supplement: Supplementary file 1 [file biomolecules-10-01239-s001.pdf]

# Supplementary Materials

## N-terminal segment of *Tv*CyP2 cyclophilin is involved in self-association, membrane interaction and subcellular localization

Sarita Aryal[1, 2, 3] Hong-Ming Hsu[4] Yuan-Chao Lou[1] Chien-Hsin Chu[1] Jung-Hsiang Tai,[1] Chun-Hua Hsu[5,*] Chinpan Chen[1,*]

[1]Institute of Biomedical Sciences, Academia Sinica, Taipei 115, Taiwan

[2]Chemical Biology and Molecular Biophysics, Taiwan International Graduate Program, Academia Sinica. Taipei 115, Taiwan

[3]Department of Chemistry, National Tsinghua University, Hsinchu 300, Taiwan

[4]Department of Tropical medicine and parasitology, National Taiwan University, Taipei 106, Taiwan

[5]Department of Agricultural Chemistry, National Taiwan University, Taipei 106, Taiwan

[*]**Corresponding authors:**

Chinpan Chen

E-mail: bmchinp@ibms.sinica.edu.tw

Professor

Institute of Biomedical Sciences, Academia Sinica, Taipei 115, Taiwan

Chun-Hua Hsu

E-mail: andyhsu@ntu.edu.tw

Professor

Department of Agricultural Chemistry, National Taiwan University, Taipei 106, Taiwan

**Table S1.** Comparison of interactions among N-terminal segment with *Tv*CyP2, Myb1$_{104-111}$ peptide with *Tv*CyP1 (PDB ID 5YBA) and CsA with *h*CyPA (PDB ID 1CWL) according to the X-ray structural data. Data were analyzed and interpreted using software tools *LigPlot, Discoveries studio* and *PyMOL*.

| **Types of Interaction** | ***Tv*CyP2_N-terminal segment** | | ***Tv*CyP1_Myb1 peptide** | | ***h*CyPA_Cyclosporine A** | |
|---|---|---|---|---|---|---|
| | N-terminal segment | *Tv*CyP2 active site | Myb1 peptide | *Tv*CyP1 active site | Cyclosporine A | *h*CyPA active site |
| **H-bonds** | R8 :O | R75:NH2 | P107:O | R63:NH2 | MeLeu3:O | R55:NH2 |
| | I10:O | Q83:NH2 | Y105:O | Q71:NH2 | MeLeu3:O | R55:NH2 |
| | S11:NH | N122:O | G106:NH | N110:O | MeBmt5:O | Q63:NH2 |
| | S11:O | Q131:NH2 | Y105:OH | T115:O | ABU6:NH | N102:O |
| | T7:O | W141:NH | K108:O | W129:NH | MeLeu2:O | W121:NH |
| **Hydrophobic** | A6 | F80 | W109 | Q67 | MeLeu2 | F60 |
| | T7 | L142 | K108 | H133 | MeVAL4 | M61 |
| | R8 | H146 | P107 | F68 | MeVAL4 | F113 |
| | V9 | M81 | P107 | M69 | MeVAL4 | L122 |
| | V9 | A121 | P107 | A109 | MeVAL4 | H126 |
| | V9 | F133 | P107 | F121 | MeBmt5 | A103 |
| | V9 | H146 | P107 | L130 | ABU6 | G72 |
| | V9 | L142 | P107 | H134 | ABU6 | A101 |
| | S11 | G92 | Y105 | G80 | ABU6 | Q111 |
| | S11 | Q131 | Y105 | A111 | | |
| | P13 | Y93 | Y105 | Q119 | | |

**Figure S1**

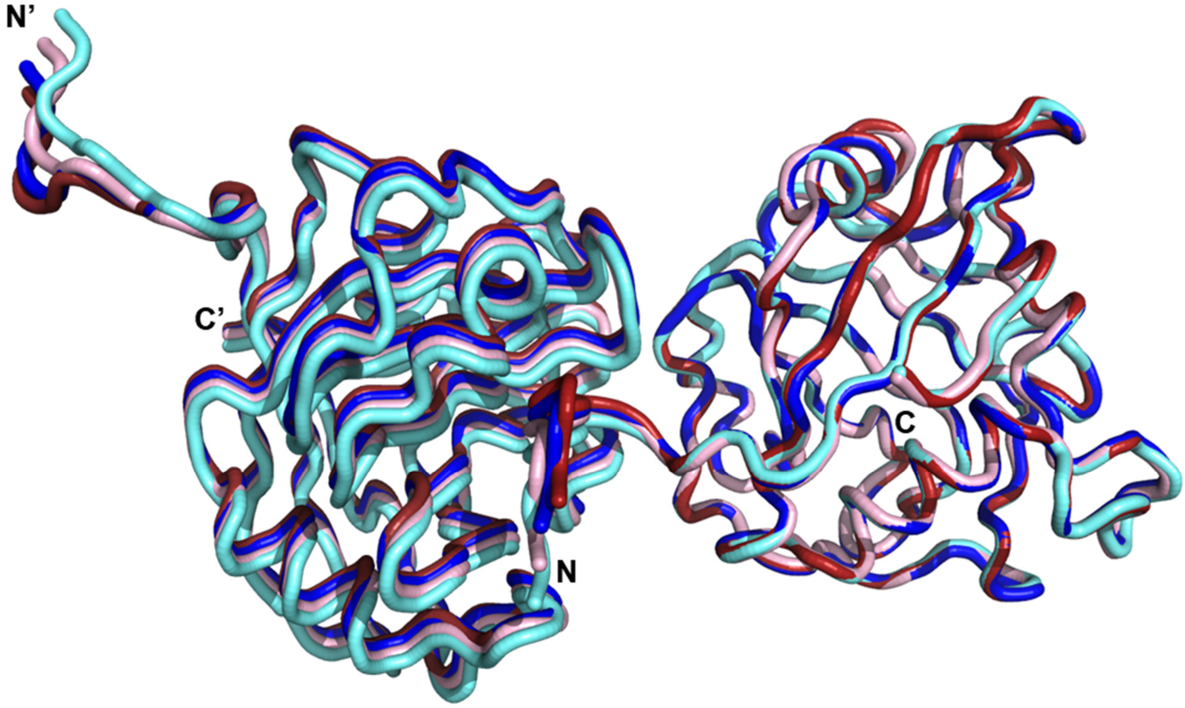


**Figure S1**. Structural superimposition of four X-ray structures, *Tv*CyP2_apo1 (cyan), *Tv*CyP2_apo2 (pink) *Tv*CyP2_apo3 (red) and *Tv*CyP2_apo4 (blue), determined under different conditions.

## Figure S2

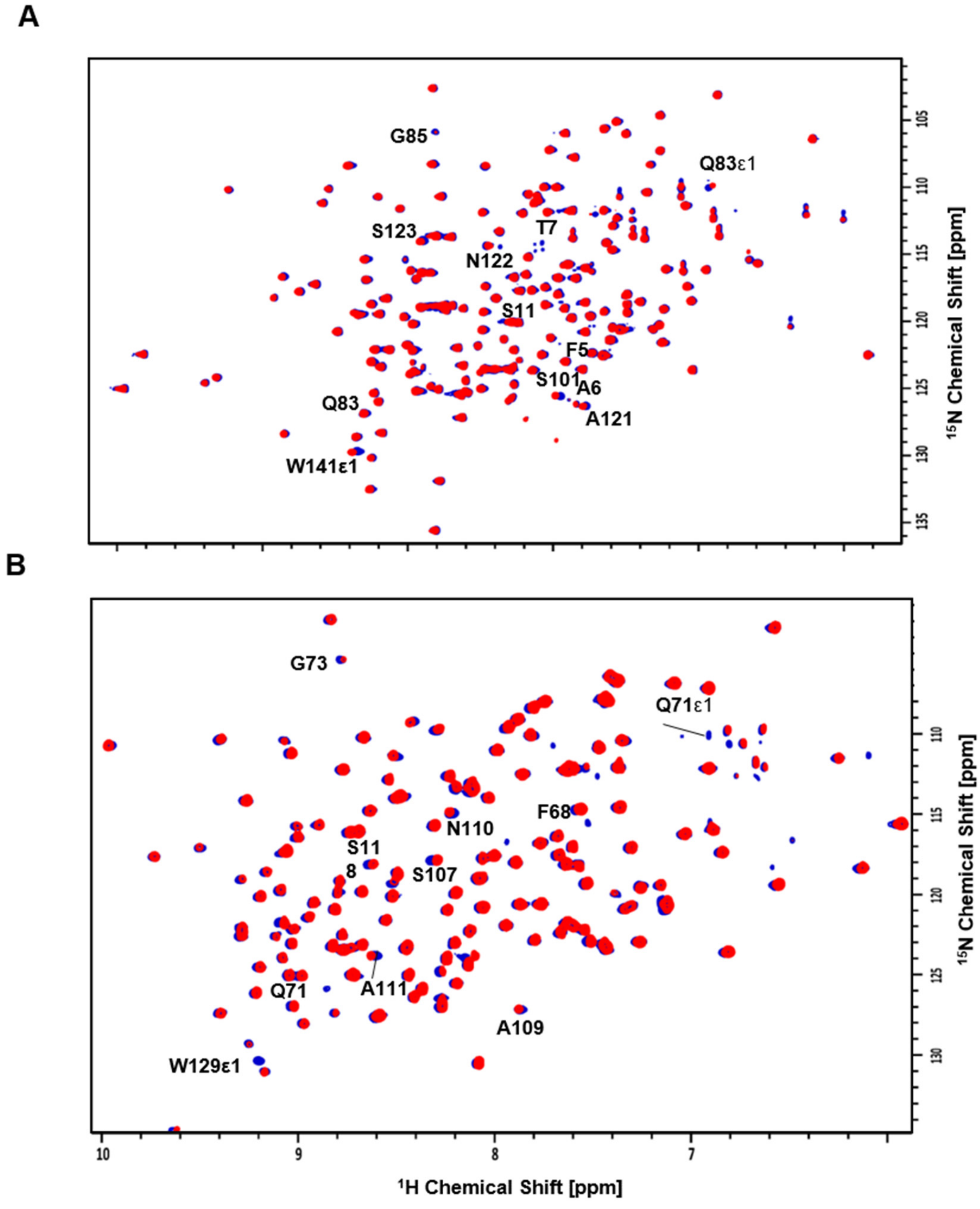


**Figure S2.** (A) 2D $^{1}H$-$^{15}N$ HSQC spectra for *Tv*Cyp2 in blue overlapped with that for *Tv*Cyp2 titrated with unlabeled *Tv*CyP2$_{3\text{-}18}$ (1:5 molar ratio) shown in red. (B) 2D $^{1}H$-$^{15}N$ HSQC spectra for *Tv*Cyp1 in blue overlapped with that for *Tv*Cyp1 titrated with unlabeled *Tv*CyP2$_{3\text{-}18}$ (1:5 molar ratio) shown in red. Cross peaks that showed chemical

shift perturbation or line width broadening upon adding the *Tv*CyP2$_{3\text{-}18}$ peptide are labelled.

**Figure S3**

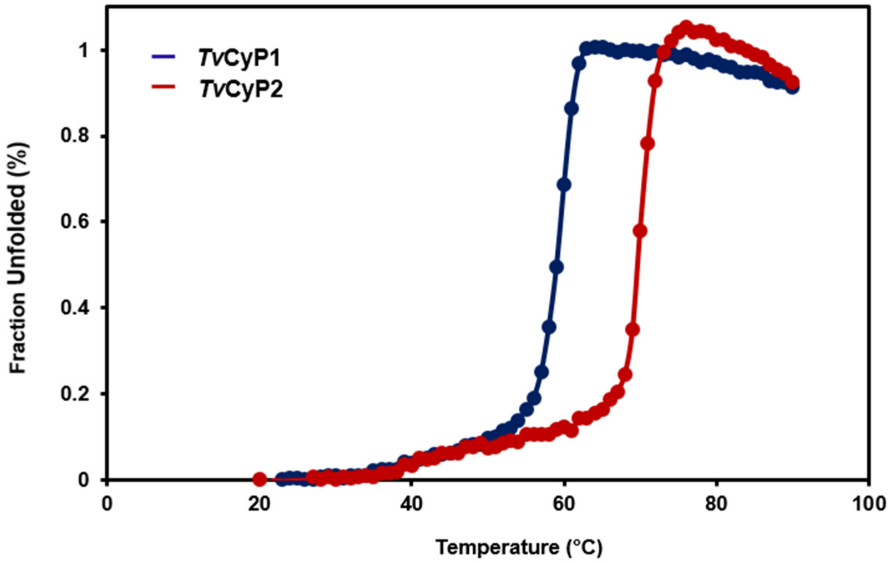


**Figure S3.** Thermal unfolding CD spectra of *Tv*CyP2 and *Tv*CyP1 shown in red and blue, respectively, from 20 to 90 °C at wavelength 222 nm. *Tv*CyP2 with $T_m$ 70°C was more stable than *Tv*CyP1 with $T_m$ 60°C.

**Figure S4**

**A**

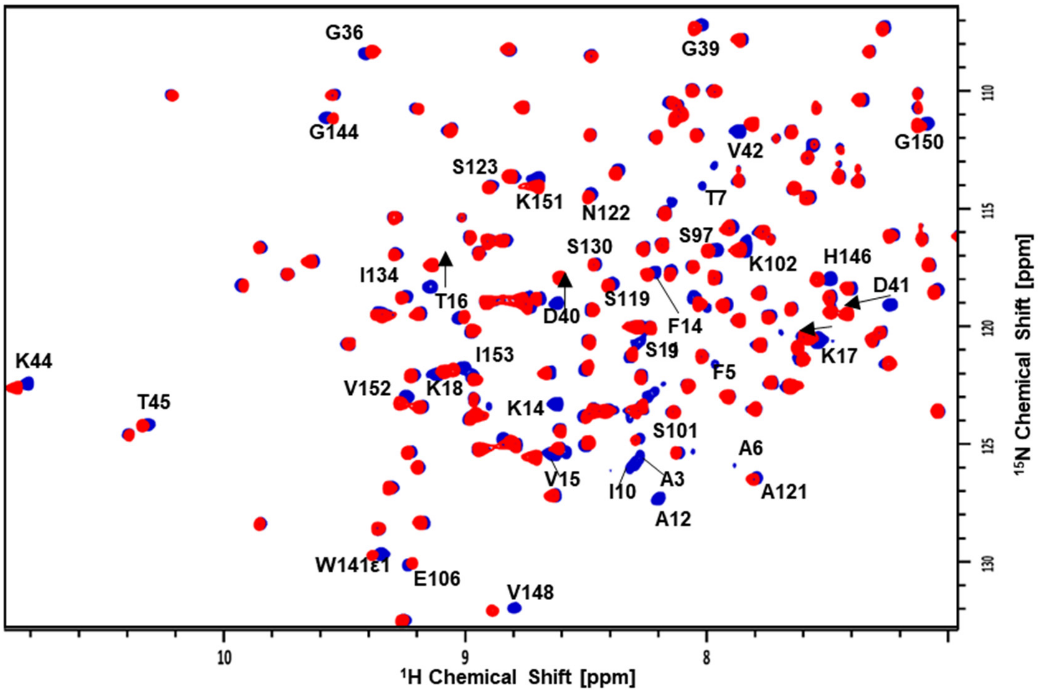


**B**

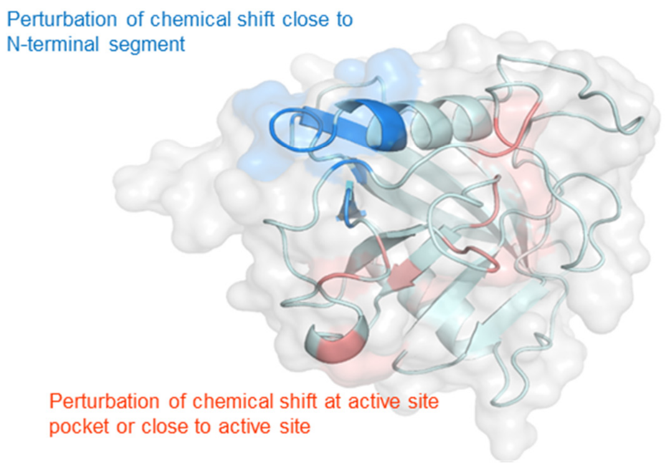


**Figure S4.** (A) The superimposed 2D $^{1}H$-$^{15}N$ HSQC spectra between *Tv*Cyp2 in blue and *Tv*Cyp2-ΔN in red. Residues showing chemical shift perturbation between the two spectra and disappeared in *Tv*Cyp2-ΔN due to the lack of N-terminal segment are annotated. (B) Residues with shift perturbations are mapped onto the structure,

showing they are close to the N-terminal segment (blue) or in the active-site pocket and its nearby region (red).

**Figure S5**

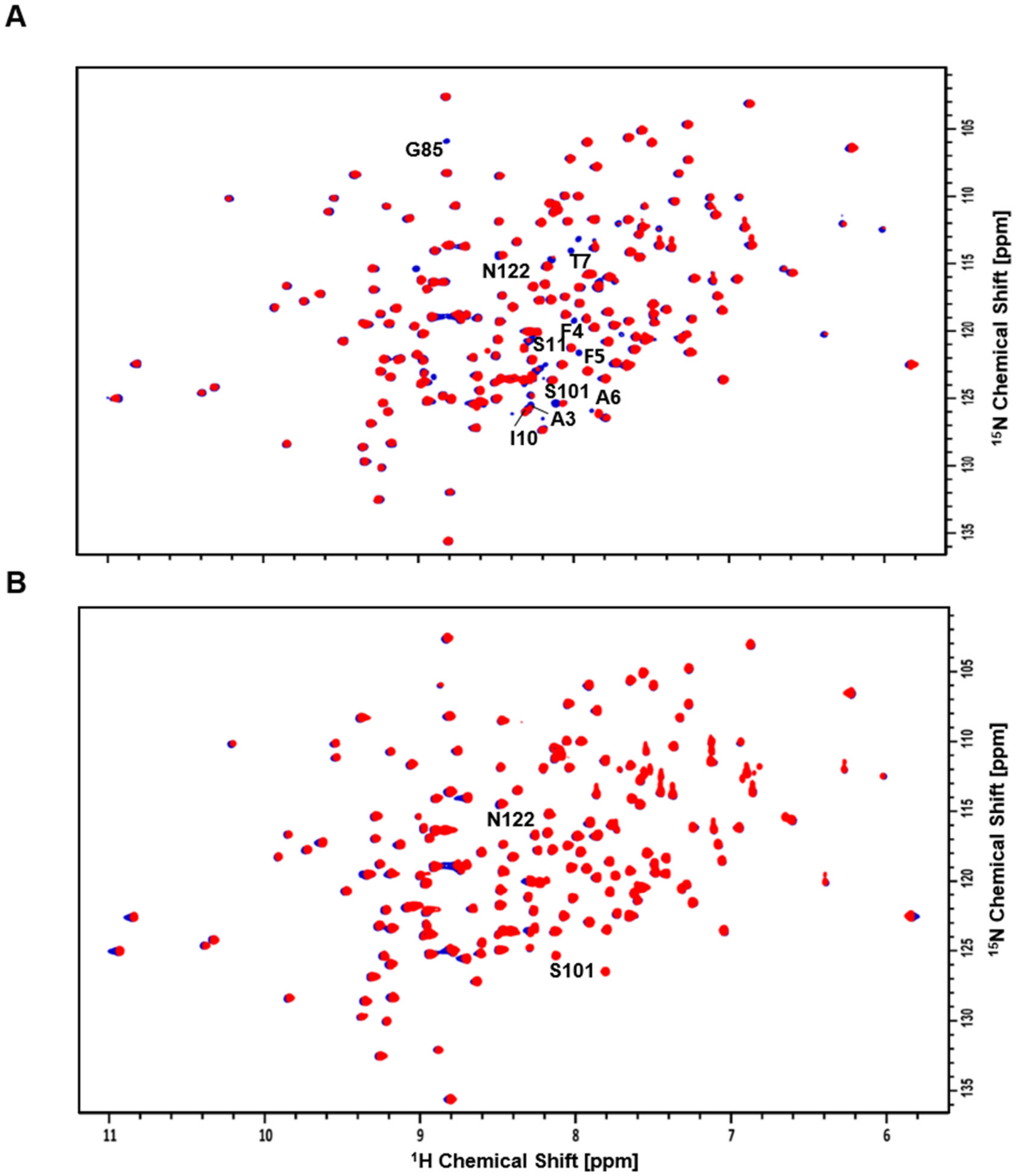


**Figure S5.** (A) The overlapped 2D $^{1}$H-$^{15}$N HSQC spectra of *Tv*CyP2 in aqueous solution (blue) and in the presence of DPC micelles (red). Cross peaks with shift perturbation, line width changing, and disappeared are labelled. (B) The overlapped

2D $^{1}H$-$^{15}N$ HSQC spectra of *Tv*CyP2-◎N in aqueous solution (blue) and in the presence of DPC micelles (red). Nearly all cross peaks are identical, which indicates no interaction between *Tv*CyP2-◎N and DPC micelles.

**Figure S6**

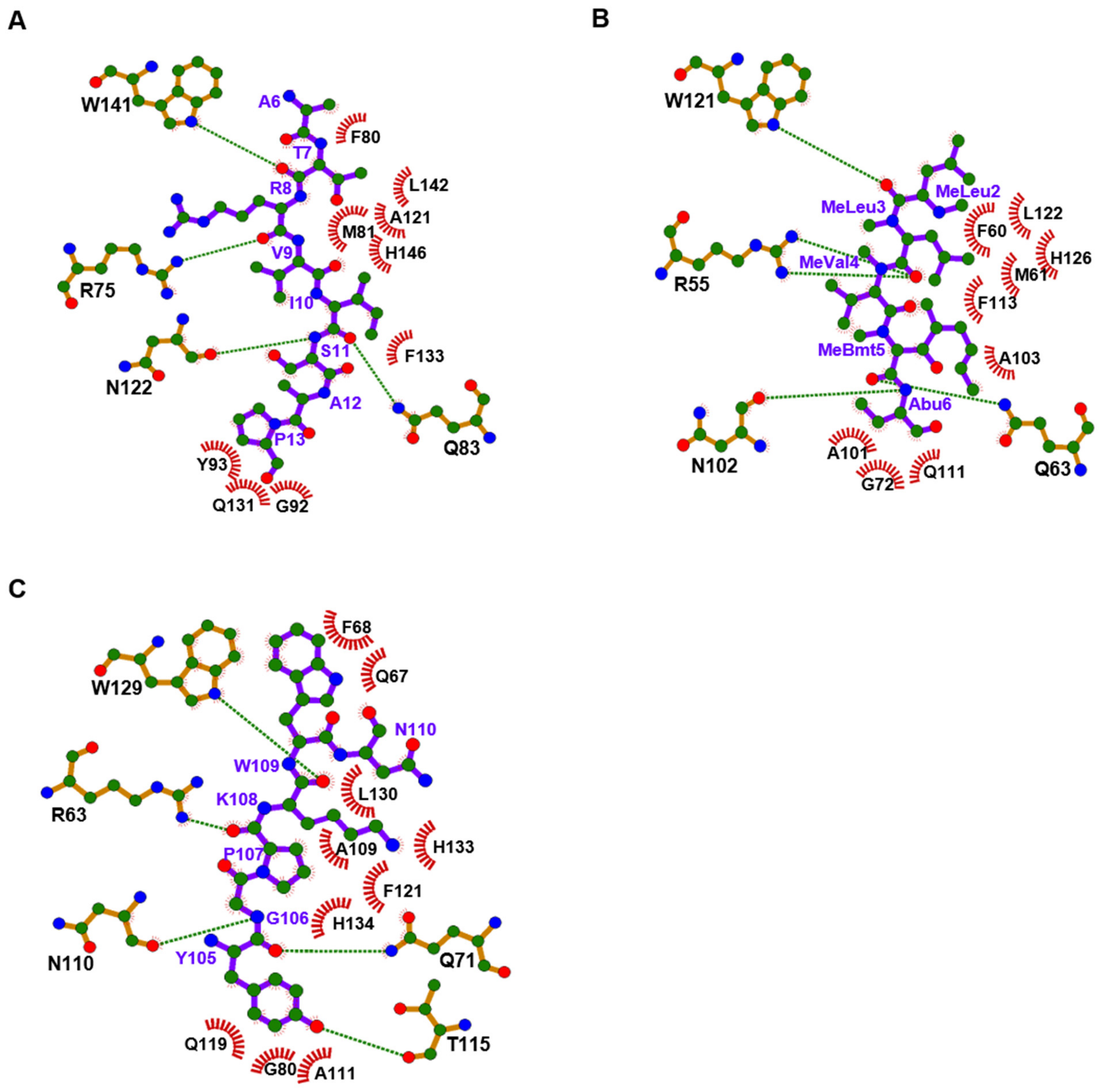


**Figure S6.** (A) H-bond and hydrophobic interactions observed between N-terminal segment and a neighboring *Tv*CyP2. (B) H-bond and hydrophobic interactions observed in *h*CyPA–CsA complex. (C) H-bond and hydrophobic interactions

observed in *Tv*CyP1–Myb1$_{105\text{-}110}$ complex. All figures were generated by LigPlot+, with H-bonds indicated by green dashed lines and hydrophobic interactions by red spokes. Carbon atom is shown in green, oxygen in red and nitrogen in blue.
